# Symptomatic Meckel's Diverticulum in Pediatric Patients—Case Reports and Systematic Review of the Literature

**DOI:** 10.3389/fped.2019.00267

**Published:** 2019-06-26

**Authors:** Daniel Keese, Udo Rolle, Stefan Gfroerer, Henning Fiegel

**Affiliations:** Department of Pediatric Surgery, University Hospital Frankfurt, Goethe-University Frankfurt am Main, Frankfurt, Germany

**Keywords:** Meckel's diverticulum, children, complications, case reports, surgery

## Abstract

**Introduction:** Our aim was to highlight the characteristics of pediatric Meckel's diverticulum with a special focus on its complications.

**Methods:** We report a group of seven patients with Meckel's diverticulum and its resection from the Department of Pediatric Surgery between 2012 and 2017. We reviewed all patient records, clinical presentation, and intraoperative findings. The diagnosis was confirmed by surgery and pathology. For a systematic literature review, we used PubMed, Medline and Google Scholar search engines to locate articles containing terms such as Meckel's diverticulum, children, pediatric, complications and symptomatic. We included article reporting on case series in English and German on pediatric patients only.

**Results:** All included patients (*n* = 7) were symptomatic. Some patients showed isolated symptoms, and others presented with a combination of symptoms that consisted of abdominal pain, bloody stool or vomiting. The median age of our seven cases was 3.5 years, including 4 male and 3 female patients. Intestinal obstruction was the most common complication; it was seen in 5 out of 7 patients (intussusception in 4 cases, volvulus in 1 case). Ectopic gastric tissue was identified in 3 cases, and inclusion of pancreatic tissue was observed in 1 case. The literature review identified 8 articles for a total of 641 patients aged between 1 day and 17 years and a male:female ratio of 2.6:1. From this group, 528 patients showed clinical symptoms related to Meckel's diverticulum. The most common symptom was abdominal pain and bloody stool. The most common surgical finding in symptomatic patients was intestinal obstruction (41%), followed by intestinal hemorrhage (34%). Complications such as perforation (10%) and diverticulitis (13%) were less frequently reported. Heterotopic tissue was confirmed on histopathology in 53% of all patients enclosing gastric, pancreatic, and both gastric and pancreatic mucosae. In one case, large intestine tissue could be found. Overall, one death was reported.

**Conclusion:** The presented case series and literature review found similar clinical presentations and complications of Meckel's diverticulum in children. Intestinal obstruction and bleeding are more frequent than inflammation in pediatric Meckel's diverticulum. Bowel obstruction is the leading cause for complicated Meckel's diverticulum in patients younger than 12 years.

## Key Points

1. Summarize of the established knowledge on this subject.
  - About 2% of infants are born with a MD.
  - It is important to maintain a high suspicion of MD in pediatric patients with.
  - Symptoms of abdominal pain and hemorrhage.
  - Management of symptomatic MD involves surgical removal, and early diagnosis and timely operative intervention can provide the best outcome.
2. What are the significant findings of this study?
  - Bowel obstruction is the leading cause of a complication in MD in children.
  - Younger than 12 years.
  - Heterotopic tissue is one of the main causes of complicated MD.
  - The awareness of the differential diagnosis of MD in children is ongoing.

## Introduction

Meckel's diverticulum (MD) was initially identified by Hildanus in 1598 and reported by Johann Friedrich Meckel, who established its embryological origin in 1809. It comprises the three layers of the intestinal wall and therefore is a true diverticulum that results from an incomplete obliteration of the omphalomesenteric duct (1). The duct typically obliterates during the 7 month of gestation, and failure of closure results in a diverticulum 98% of the time (2). Due to the embryological origin, MD is almost always located within 2 feet of the ileocaecal valve on the anti-mesenteric border at the terminal ileum and rarely on the mesenteric side. It is the most prevalent congenital anomaly of the gastrointestinal tract, occurring in 2% of the population with a 2:1 male predominance. Approximately 2% of patients develop complications over the course of their lives, typically before the age of 2 years (2, 3). Patients are usually asymptomatic, but they can present with abdominal pain, gastrointestinal bleeding and bowel obstruction or diverticulitis with or without intestinal perforation (4). Histological MD can contain heterotopic rests of gastric mucosa, which is seen in 50–60% of cases and may cause abdominal pain, ulceration and bleeding (5). Duodenal, colonic or pancreatic tissue has been identified in 5–6% of MD cases and in most cases was responsible for intestinal obstruction (6). With its varied presentations, MD often becomes a diagnostic challenge (7). Especially in children, symptomatic MD can be easily misdiagnosed, which is why pediatricians and pediatric surgeons should be well aware of its possible presentations (8).

The following illustrative presentation shall outline the different clinical presentation forms of MD based on our experience in cases evaluated from 2012 to 2017 in our department of pediatric surgery. We additionally highlight three cases of children with the following complications: intussusception in an 8-week-old female infant, a 3.5-year-old boy with painless hemorrhage and a 3-year-old boy with a volvulus. Furthermore, we review the literature on MD and its forms of clinical presentation in children.

## Materials and Methods

**Figure 1:**
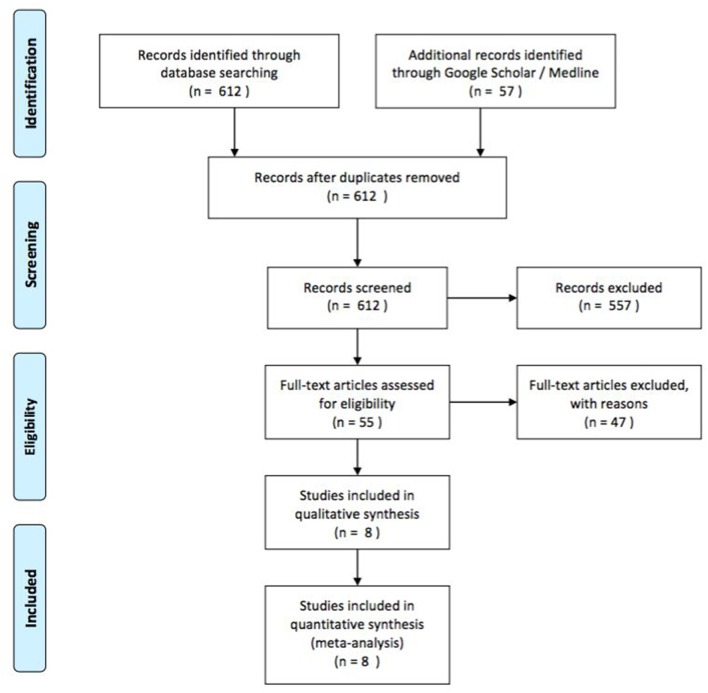
Flow chart showing the results of the literature search.

We present a series of seven cases of MD in children hospitalized in our Clinic of Pediatric Surgery (University Hospital Frankfurt, Germany) from January 2012 to January 2017. Out of these seven cases, we highlight three cases that involved complications such as intussusception, hemorrhage and volvulus. Medical records were reviewed retrospectively including clinical presentation at admission, laboratory values, performed preoperative diagnostics, intraoperative findings, and histological results. For a systematic review of the literature, we used PubMed, Medline and Google Scholar search engines for articles containing terms such as “Meckel's diverticulum,” “children” AND/OR “pediatric” AND “complications” AND/OR “symptomatic.” We included those articles reporting on case series and case reports in the English and German languages and selected articles for further reading based on the title and abstract. Studies with patients older than 17 years of age were excluded as well as those reports on patient series with <20 symptomatic patients (quantitative screening). Further eligibility criteria were the report of clinical presentation, sex and age, operation as treatment, histology of the MD and location (qualitative screening). Data for all clinical, laboratory, radiological, intraoperative and pathological findings were collected from the different databases. We created a PRISMA flow chart showing the results of the literature search (Figure 1).

### Case Reports

#### Case 1: Intussusception in the Youngest Patient

**Figure 2:**
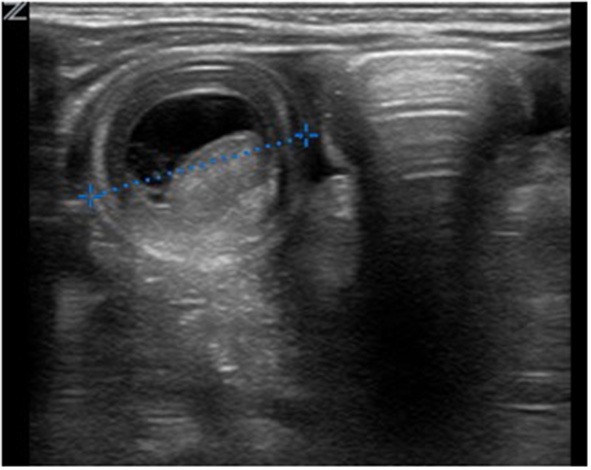
Sonography in the transverse plane of the right lower abdominal quadrant revealed the typical “target sign” of intussusception with a diameter of 27 mm, focal bowel wall thickening and fluid between the two bowel walls.

**Figure 3:**
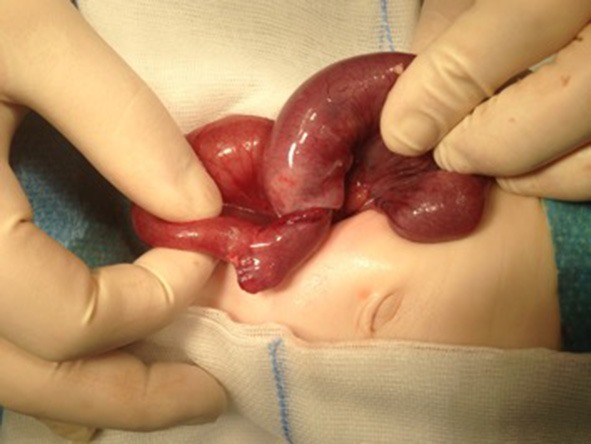
Ileoileal intussusception with Meckel's diverticulum, which acts as a lead point lesion for the intussusception.

An 8-week-old female (39th week of pregnancy; birth weight: 2600 g) infant was admitted to our emergency department with subfebrile temperatures up to 37.7°C, partially bilious vomiting and increasing listlessness, with refusal to eat for the previous 24 h. Her last defecation (non-bloody) was noticed 3 days prior to admission. Except for abdominal distension and hypoactive bowel sounds, no other abnormal general examination findings were apparent. Laboratory parameters showed an elevated CRP of 4 mg/dl. The ultrasound presented an intussusception in the right lower abdomen. Hydrostatic reduction with sodium chloride 0.9% was performed under low sedation using midazolam (0.1 mg/kg body weight). The infant was rehydrated overnight, and vomiting was suspended. Another ultrasound showed significant dilation of the bowel loops in the right lower abdomen, free fluid and typical signs of intussusception (Figure 2). Two more reduction attempts were made without success; thus, the infant was taken for laparotomy. During surgery an ileoileal intussusception 25 cm from the ileocolic junction was observed including ischaemic changes. On reduction, a typical MD with a size of 1 cm was identified more proximal to the ileocolic valve acting as a lead point lesion for intussusception (Figure 3). A 10-cm necrotic ileum segment with the MD was resected with primary anastomosis. The infant received antibiotics (ceforuxim/metronidazole) for 5 days postoperatively. Histological examination revealed the presence of an MD containing ectopic pancreatic tissue. The ileum showed mucosal necrosis, ulceration and infarction accompanied by a fibropurulent peritonitis.

#### Case 2: Painless Hemorrhage

A 3-year-old boy presented with a 3-day history of painless rectal bleeding (dark red) with no other symptoms. His past medical history was unremarkable. The initial examination showed a stable patient with a normochromic, microcytic anemia with a hemoglobin level of 7.1 g/dl. Biochemical assessments of liver and renal functions were normal. Digital rectal examination was unremarkable. Gastroduodenoscopy and colonoscopy showed no bleeding source. After i.v. treatment with omeprazole (20 mg), blood samples presented no further decrease in hemoglobin. The patient was always haemodynamically stable. The next day, we performed a diagnostic laparoscopy. Intraoperatively a 2 × 3-cm MD was found, approximately 25 cm proximal to the ileocaecal valve. An ileal segmental resection with 5.0 cm of small bowel including the MD was performed through a limited subumbilical laparotomy. The pathology report described an MD with ectopic gastric mucosa and an inflammatory reaction. Postoperatively, the patient first received imipenem i.v. according to the microbiological results. Six days after surgery the patient had recovered without incident and was discharged from the hospital.

#### Case 3: Volvulus

**Figure 4:**
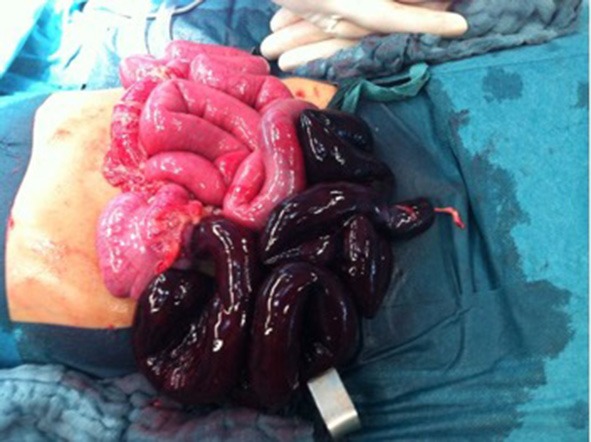
The cause of the volvulus is a Meckel's diverticulum with the omphaloenteric duct is shown. The necrotic part of the ileum is approximately 50 cm long and reaches the ileocaecal junction.

A 3-year-old previously healthy boy presented with acute onset of abdominal pain and vomiting (not bilious) for the previous 12 h. His last defecation the previous day was normal. On physical examination, the patient was somnolent and tachycardiac; all other vital signs were stable. He had abdominal distension without ubiquitous tenderness. Bowel sounds were decreased, and a digital rectal examination showed bloody marks on the examining finger. Routine laboratory showed elevated inflammation parameters (CRP: 4.38 mg/dl) and a hyponatraemic acidosis. Abdominal ultrasound revealed free fluid in the right lower abdomen and dilatated intestinal loops with a 2.6 cm diameter and aperistalsis. The patient was taken to the operating room and underwent laparotomy with the finding of a midgut volvulus caused by an MD with the omphalomesenteric duct 30 cm proximal to the ileocaecal valve (Figure 4). A total of 50 cm of gangrenous bowel was resected with primary anastomosis. Additionally, an appendectomy was performed. Histology revealed necrotic small bowel with a broad-based MD including mucus-producing goblet cells. The patient's postoperative course was entirely unremarkable. He received cefuroxim/metronidazole as the antibiotics and was discharged on the eighth hospital day with normal defecation and standard blood values.

## Results

A total of 7 patients had surgery for MD in our Department of Pediatric Surgery from 2012 to 2017; 4 patients (57%) were male, and 3 patients (43%) were female. The median age of presentation was 3.5 years. The oldest patient observed was 16 years of age, and the youngest patient was 8 weeks old. Intestinal obstruction was the most common complication observed in 5 out of 7 patients (71%) resulting from intussusception (4 of 5) and volvulus (1 of 5) (Table 1). Patients with intussusception were younger than 6 months or older than 2 years of age. Clinically, the 5 patients with obstruction presented with abdominal pain, vomiting and abdominal distension. Two of the 7 patients had a hemorrhage, 1 without pain, the other with abdominal pain. All 7 patients received routine investigations including blood work and ultrasound. In one patient with a hemorrhage, we performed an oesophagogastroduodenoscopy (EGD). After stabilization, all patients underwent primary laparotomy or explorative laparoscopy followed by a laparotomy. The MD was typically located between 25 and 35 cm proximal to the ileocaecal valve. A remnant of the vitelline duct was present in 1 case. Histopathological investigation confirmed the diagnosis of MD in all seven cases containing ileal mucosa. Ectopic tissue was found in 5 cases (71%); gastric tissue was identified in 3 cases (43%), including those presenting with a hemorrhage. In 1 case, inclusion of pancreatic tissue was observed (14%), and in 2 other cases, hypertrophic lymphoid follicles in the MD wall were observed. The number of days in the hospital until discharge varied from 7 to 9 days.

**Table 1:** Summary of our seven cases.

**Age**	**Gender**	**Presenting cardinal symptome**	**Performed preoperative diagnostics**	**MD complications**	**Length of MD**	**Histology**	**Length of stay (days)**
2 m	Female	Bilious vomiting	Ultrasound	Intussusception	1.0 cm	Ileal mucosa + pancreatic tissue	8
4 m	Male	Bilious vomiting	Ultrasound	Intussusception	1.5 cm	Ileal mucosa + ectopic gastric tissue	9
3.5 y	Female	Abdominal pain	Ultrasound	Intussusception	1.5 cm	Ileal mucosa + ectopic gastric tissue + hyper-trophic lymphoid follicles	7
3.5 y	Male	Hemorrhage	EGD	Bleeding	0.8 cm	Ileal mucosa + ectopic gastric tissue	7
3.5 y	Male	Abdominal pain	Ultrasound	Volvulus	1.5 cm	Ileal mucosa	7
9 y	Female	Abdominal pain	Ultrasound	Intussusception	2.0 cm	Ileal mucosa	7
16 y	Male	Abdominal pain/Hemorrhage	Ultrasound	Bleeding	1.5 cm	Ileal mucosa + ectopic gastric tissue	8

*EGD, esogastroduodenoscopy; y, years; m, months*.

The literature search results are shown in Table 2. After reviewing and complying with the inclusion criteria, 8 articles were listed. In these articles, a total of 641 patients were evaluated; 464 male and 177 female pediatric patients were diagnosed with MD, with a male:female ratio of 2.6:1. The age range was from 1 day to 17 years. Those authors who reported about a mean age showed values between 3.2 years (4) and 5.3 years (2). Three case series did not provide individual patient ages. Stanescu et al. distributed their patients into childhood periods aged between 0 and 16 years with no information about the mean age or a median. Blevrakis reported a median patient age of 10 years but reports less information about the distribution. Menezes also had limited information about the patients age. The review shows a range from 2 days to 14 years but gives no exact distribution or a mean age. Clinical symptoms were reported in 528 cases. Clinical manifestations of MD were various in nature. The most common manifestations reported in all articles were abdominal pain and gastrointestinal bleeding/bloody stool. Fever, tachycardia or restlessness was described in 3 series that are described as general symptoms in Table 3. Vomiting and nausea were reported explicitly in 5 articles. Except for 2 series, all other authors included patients without clinical symptoms. In all of these patients, MD was incidentally diagnosed during surgery for other diseases (12%); thus 82% of all patients had surgery for symptoms related to their MD. In this symptomatic group, 53% of all patients were ≤ 4 years of age. In 3 studies, no diagnostic management was described. Abdominal ultrasound was used in all other studies for preoperative diagnosis. In addition, X-ray and/or CT scans were utilized in individual cases. The most common surgical finding in symptomatic patients was intestinal obstruction with 41% of cases (variation between 14 and 86%) occurring as a result of MD. In 17% of those patients (reported in 6 series), intussusception was explicitly reported (variation between 9.5 and 35%), and in 14% of patients, a volvulus was described as intestinal obstruction (variation between 7 and 18%; reported in 3 series). Other reasons such as an intestinal band, hernia, kinking, knotting of the gut, general intestinal obstruction and/or adhesions were reported in 23% of patients (variation between 8.5% and 52%). The second most common complication was an intestinal hemorrhage (34% of all symptomatic patients) with a variation of 4.6 to 44%. Complications such as perforation (10%, reported in 6 series) and diverticulitis (13%, reported in all series) were less frequent (Table 4). Heterotopic tissue was confirmed on histopathology in 53% of all patients (including patients with incidentally detected MD), with a variation between 7.7 and 73%. Out of these results, gastric, pancreatic, and both gastric and pancreatic mucosae were reported in 41% (4.6–61%), 8.8% (2–34%), and 4.6% (2–10%) of patients, respectively. In 1 patient, tissue of the large intestine was identified (0.2%) (Table 5). When highlighting the symptomatic group, 59% of the resected MD showed ectopic tissue. All patients of the symptomatic group (528) underwent resection of the MD. In total, 30 patients (5.6%) presented complications after surgery. Postoperative ileus was the main indication for reoperation; wound infection and paraumbilical hernia were less frequent. Overall, 1 death was reported. This patient presented late with perforation of an MD with septic shock and diffuse peritonitis. The mortality rate was thus 0.2% (Table 6).

**Table 2:** Systematic literature review—patients data.

**Series**	**Stanescu**	**Gezer**	**Rattan**	**Lin**	**Blevrakis**	**St-Vil**	**Menezes**	**Huang**
Total patients	44	50	65	102	45	164	71	100
No. of symp. patients	29	42	65	92	20	117	63	100
Male:Female	33:11	36:14	52:13	65:37	32:13	120:44	52:19	74:26
Ave. age (years)	0–16	4.6	3.2	5.6	1–10	5.2	2d−14y	5.3

**Table 3:** Systematic literature review—clinical symptoms.

**Symptoms**	**Stanescu**	**Gezer**	**Rattan**	**Lin**	**Blevrakis**	**St-Vil**	**Menezes**	**Huang**
Abdominal pain	+	+	+	+	+	+	+	+
*Peritonitis*				+				
Nausea/Vomiting	+	+	+			+		+
*Constipation*			+					
Umbilical pathology			+			+		
*General Symptoms*	+							+
GI-Bleeding	+	+	+	+	+	+	+	+

**Table 4:** Systematic literature review—complications.

**Complications**	**Stanescu**	**Gezer**	**Rattan**	**Lin**	**Blevrakis**	**St-Vil**	**Menezes**	**Huang**
**Intestinal obstruction**	14 (48.3%)	16 (38.1%)	56 (86.2%)	39 (42.3%)	4 (20%)	49 (41.8%)	9 (14.3%)	41 (41%)
-Intussusception		8 (19%)	12 (21.4%)	32 (34.8%)		19 (16.2%)	6 (9.5%)	17 (17%)
-Volvulus		3 (7.1%)	10 (17.8%)			20 (17.1%)		
- Others			34 (52.3%)^*^	7 (7.6%)^*^		10 (8.5%)^*^		24 (24%)^*^
**Intestinal hemorrhage**	7 (24.1%)	14 (33.3%)	3 (4.6%)	41 (40.2%)	7 (35%)	45 (38.5%)	35 (56%)	44 (44%)
Perforation		5 (11.9%)	4 (6.1%)	8 (7.8%)	3 (15%)	10 (8.5%)	6 (9.5%)	
Diverticulitis	8 (27.6%)	5 (11.9%)	2 (3.1%)	4 (3.9%)	6 (30%)	2 (1.7%)	4 (6.3%)	15 (15%)

* *others: band, hernia, kinking, knotting of gut, general intestinal obstruction, adhesion*.

**Table 5:** Systematic literature review—histological results.

**Mucosal Type**	**Stanescu**	**Gezer**	**Rattan**	**Lin**	**Blevrakis**	**St-Vil**	**Menezes**	**Huang**
**Ectopic tissue**	15 (34.1%)	32 (64%)	5 (7.7%)	79 (77.5%)	22 (48.9%)	71 (50%)	49 (69%)	73 (73%)
Gastric	11 (25%)	26 (52%)	3 (4.6%)	42 (41.2%)	18 (40%)	62 (43.7%)	43 (60%)	61 (61%)
Pancreatic	3 (6.8%)	3 (6%)	2 (3.1%)	35 (34.3%)	3 (6.6%)	5 (3.5%)	6 (8.4%)	2 (2%)
Gastric and pancreatic		3 (6%)		2 (2%)	1 (2.2%)	4 (2.8%)		10 (10%)
Large intestine	1 (2.3%)							

**Table 6:** Systematic literature review—morbidity and mortality.

	**Stanescu**	**Gezer**	**Rattan**	**Lin**	**Blevrakis**	**St-Vil**	**Menezes Huang**
Morbidity	-	12 (24%)	4 (6.1%)	2 (2.2%)	-	12 (7.3%)	-
Mortality	-	-	1 (1.5%)	-	-	-	-
							

## Discussion

The omphalomesenteric duct connects the yolk sac to the intestinal tract during early fetal life and is usually obliterated by the 7th week of gestation (1). Failure to regress can result in various anomalies including MD, patent vitelline duct, fibrous band, sinus tract, umbilical polyp and umbilical cyst. Of these anomalies, MD is the most common and can occur in 2% of the general population (8). According to the rule of “2s”, the MD is located at a distance of 2 feet from the ileocaecal valve, usually is 2 inches in length and 2 cm in diameter and is twice as common in males than in females. Two-thirds of MD specimens have ectopic mucosae. The two most ectopic tissues reported are gastric and pancreatic tissues. A total of 2% of patients become symptomatic with diverse symptoms, which can make the diagnosis difficult (4). There is no familial predisposition for MD, but the prevalence is increased in children with other serious diseases such as neoplasm, intestinal obstruction, omphalocele, gastroschisis or malrotation (9). Although most of the MD cases are asymptomatic, it can give way to different clinical presentations and complications. Most of the different case reports and studies include both pediatric and adult patients (10, 11). Thus, our objective was to conduct an observational, retrospective and descriptive study to highlight the most common presentation form of symptomatic MD in children.

### Clinical Presentation

A large proportion of MD cases in children occur before the age of 2 years, and between 25 and 50% of patients with symptoms present younger than 10 years of age (12). In our report, the average age presentation of 5.1 years was similar to the results of the reviewed studies, as was the male:female ratio, which has been reported as between 2:1 to 4:1 (2–4, 13). The clinical manifestation of MD has been found to be variable, with little specificity. The majority of patients in all reported series presented with abdominal pain that was mostly localized around the umbilicus, in the right iliac fossa or in the lower abdominal area. Additionally, patients presented with bloody stool, followed by nausea and vomiting (1, 3, 13–15). Two authors described general symptoms, including fever, dyspnoea or neurological disorders (2, 3). Symptomatic MD occurred in 4.2 to 16.9% of individuals with MD (16). Painless gastrointestinal bleeding and obstruction were the most common complications in children (2).

### Intestinal Obstruction

There are various forms of intestinal obstruction resulting from intussusception, omphalomesenteric bands (as the result of persisting umbilical attachments) or adhesions and accounts for 26–53% of complications (3). Most studies report intestinal obstruction as the most common complication in the pediatric age group (4, 9, 12). Some authors such as Blevarakis et al and Menezes et al. reported a very low incidence (8.9 and 14.2%, respectively) of intestinal obstruction (13, 14). In children, intussusception and volvulus appear to be the most common etiology of intestinal obstruction, whereas in adults, these findings are uncommon (10, 11). In our reported series, the most common form of obstruction was summarized into the category of general obstruction including intestinal band, hernia, kinking, knotting of the gut, or adhesions. Despite the fact that 2 authors did not describe the explicit form of obstruction, intussusception was reported as one of the most common complications of this category. Intussusception, in which the MD drops into the bowel lumen and then serves as a lead point to allow telescoping of the small intestine into first the distal ileum and then into the large intestine causes ileoileal and ileocolic types of intussusception; it should be considered in the differential diagnosis of intussusception in all patients, particularly in children younger than 6 month or older than 2 years of age (1). Nevertheless, the presenting symptoms of intussusception due to MD are non-specific, including abdominal pain as described. As reported in our abstract, while hemorrhage and obstruction are both common presentations in pediatric patients, patients with bowel obstruction seem to be younger. There are only a few reports that distinguish between the different age groups from 0 to 18 years. Patients younger than 12 years of age tend to present with obstruction with the major characteristics of patients in the age group under 4 years of age (12). Another study reports a higher incidence of bowel obstruction due to an MD in patients younger than 1 year of age compared to those older than 12 months of age. Our case reports underline this statement because all patients under 4 years of age treated in our department had bowel obstruction with intussusception.

### Gastrointestinal Bleeding

Normally, a hemorrhage is a painless symptom that typically indicates an MD in pediatric patients. Gastrointestinal bleeding presenting with bloody stool related to an MD is caused by ulceration of the small bowel due to acid secretion by ectopic mucosa within the diverticulum or rarely from adjacent ileal ulcers secondary to acid secreted in an MD. Bleeding is reported clinically as acute massive hemorrhage, anemia due to chronic bleeding, or self-limiting recurrent episodes (17). Transfusions are uncommonly reported for Meckel's-associated bleeding. Pediatric patients often present with dark red or maroon stools (18), as reported in our cases. Various case series have found that ~15% of patients with MD have ectopic tissue within the diverticulum; gastric mucosa is the most common form of ectopic tissue that causes bleeding of an MD in most cases, as seen in our review (1, 3, 13). As we described, abdominal pain is more associated with bowel obstruction. Nevertheless, both symptoms can occur at the same time, and a painful hemorrhage should not exclude an MD as it can be symptomatic for an acute abdominal disease. Thus, a hemorrhage can be seen in both intussusception and an MD especially associated with ectopic gastric mucosa.

### Perforation/Inflammation

MD perforation may also occur in approximately 10% of the symptomatic children in the first year of life (3). Inflammation is usually accompanied by fever, vomiting and abdominal pain and is often indistinguishable from acute appendicitis. Inflammation results from obstruction at the base leading to infection within the blind-ending diverticulum. Obstruction leads to bacterial overgrowth and inflammation. Perforation of an MD will manifest with signs of diffuse peritonitis, usually localized in the lower abdomen (2). In our series, out of the 7 patients with peritonitis, no one had a perforated MD, but 4 patients had diverticulitis at laparotomy. The appendix was found to be normal in 6 cases; in 1 case, the appendix showed inflammation.

### Ectopic Tissue

The lining of an MD often contains ectopic tissue, especially in patients with symptomatic MD and hemorrhage in particular (1–5). It is estimated that the incidence of symptoms in MD with ectopic tissue is as high as approximately 60%, whereas the incidence of symptoms in all MD is approximately 2-4% (2, 3). That underlines our results with 71% of ectopic tissue in our case series of symptomatic patients and 59% highlighted in the literature results. Only Rattan et al. showed low results, which were that 7.7% of 65 symptomatic patients had ectopic tissue (4) with no explanation for this discrepancy, because these results differ from all other studies with results from 41.1 to 85%. Furthermore, Burjonrappa et al. reported in their own patient series of 22 children that symptomatic pediatric patients without ectopic tissue were younger than symptomatic patients with ectopic tissue (19). Chen et al. noted that heterotopic tissue is the main cause of complicated diverticulum and suggested removal of this tissue when incidentally found in pediatric patients (20).

Complications following resection of an MD can occur. Huang et al. reported a complication rate of 5% for Meckel's resection (2). Lin et al. identified 2 patients with intestinal adhesions 2 and 7 months after discharge, respectively (15). Cullen et al. reported a morbidity of 12% for resection of symptomatic MD in adults, and the cumulative risk of long-term postoperative complications was 7% (18). The most common complications were surgical site infection, postoperative ileus, and anastomotic leak, which are essentially complications of any small bowel surgery (15). Similar to many other less common intraabdominal conditions, death frequently occurs because of delay. In contemporary practice, death that is related specifically to the resection of Meckel's diverticulum is rare, with an estimated incidence of 0.001% (4, 15). Our 7 cases showed no complications, and it is important to say that early diagnosis and timely operative intervention can provide the best outcome for these patients.

The preoperative diagnosis of a symptomatic MD is difficult because it can mimic many more common ailments that cause an acute abdomen. It is necessary to maintain a high index of suspicion in the pediatric age group (1). Initial laboratory testing should include a complete blood count, electrolytes and a coagulation test. These studies may help identify patients who are volume depleted or experiencing complications that are nonspecific and do not distinguish an MD from other anomalies. Most authors use technesium-99 pertechnetate as a diagnostic tool (4). It is necessary to say that the Meckel's scan has a poor positive predictive value and cannot be relied upon for a diagnosis in cases of a bleeding MD if the Tc99 scan is negative (4). In our 7 case reports, we only used ultrasound as a preoperative diagnostic tool. Ultrasound has increasingly become more valuable in the diagnosis of MD, as it is a tool that is non-invasive. Furthermore, it offers dynamic information, and the lack of ionizing radiation is especially important in the pediatric population in efforts to minimize radiation exposure in early life (10). In 1 case with GI bleeding, we added EGD before laparotomy. Laparoscopy should be a useful diagnostic and therapeutic tool especially in cases of a bleeding MD (1, 21). The general management of a symptomatic MD involves surgical removal. The real controversy is about how to manage asymptomatic MD. For pediatric to young adult patients, Tauro et al. suggest resection of a normal-appearing MD in every case of appendectomy or laparotomy/laparoscopy for an acute abdomen to avoid secondary complications arising from it (21). Another recent analysis of the literature suggests that resection for an incidental MD should be restricted to those identified as being at a higher risk of complications: male gender, age younger than 40 years, a diverticulum longer than 2 cm, and the presence of a macroscopic mucosal alteration noted at the time of surgery.

## Conclusion

MD represents a rare embryological remnant, which remains largely asymptomatic in childhood. MD is an important differential diagnosis in pediatric patients presenting with intestinal obstruction or GI bleeding. Clinically, abdominal pain as a result of bowel obstruction and/or inflammation and painless GI bleeding are the most common symptoms of symptomatic MD in children. Nevertheless, the diagnosis of MD still remains a challenge because of overlapping clinical and imaging features of other acute surgical and inflammatory conditions of the abdomen. Especially in children, MD should be suspected in cases of painless gastrointestinal bleeding as well as in cases of intussusception, particularly recurrent or atypical episodes. The treatment of choice for symptomatic MD in children is surgical resection, with the laparoscopic approach being the technique of choice.

Overall, the literature review and our patient data shared similar results with regard to the male:female ratio, clinical presentation and intraoperative findings as well as the histological results. With varied clinical manifestations in the pediatric age group, MD still presents a clinical and diagnostic challenge for pediatricians as well as for pediatric surgeons.

## Ethics Statement

Patients were not required to give informed consent to participate in the study because the analysis used anonymous clinical data that were obtained after each patient agreed to treatment by written consent.

## Author Contributions

All authors reviewed the manuscript and completed final approval. DK acquired and analyzed the data and wrote the manuscript draft. UR, SG, and HF contributed to study conception and design and made critical revision on the manuscript.

### Conflict of Interest Statement

The authors declare that the research was conducted in the absence of any commercial or financial relationships that could be construed as a potential conflict of interest.
